# Effects of Pregnancy and Nutritional Status on Alcohol Metabolism

**Published:** 2007

**Authors:** Kartik Shankar, Martin J.J. Ronis, Thomas M. Badger

**Keywords:** Ethanol metabolism, ethanol clearance, pregnancy, maternal alcohol exposure, fetal alcohol effects, fetal alcohol spectrum disorder (FASD), alcohol-related birth defects (ARBD), nutrition, total enteral nutrition (TEN), maternal nutrition, genetic factors

## Abstract

Metabolism of alcohol (i.e., ethanol) is regulated by genetic and environmental factors as well as physiologic state. For a given alcohol intake, the rate of alcohol clearance, which ultimately determines tissue ethanol concentrations, may be the most significant risk factor for many of the detrimental effects of alcohol. Faster ethanol clearance would help minimize target tissue concentrations, and in pregnant women, mitigate fetal alcohol exposure. Much remains to be known about the effects of the altered endocrine milieu of pregnancy on alcohol metabolism and clearance in the mother. Research has shown that among pregnant rats allowed unrestricted access to alcohol and those fed alcohol containing liquid diets under experimental conditions via a feeding tube (total enteral nutrition [TEN]), urine ethanol concentrations (and thus blood and tissue ethanol concentrations) are lower in pregnant rats compared with non-pregnant females given the same dose of ethanol. Maternal nutritional status also is an important determinant of fetal alcohol toxicity. Research using the TEN system has demonstrated that alcohol-induced fetal growth retardation is potentiated by undernutrition in part via impaired alcohol metabolism and clearance.

## Alcohol-Related Birth Defects

Although the harmful effects of alcohol (i.e., ethanol) on the growing fetus have been recognized for nearly three decades, alcohol continues to be the most common malformation-causing chemical (i.e., teratogen) ingested during pregnancy (Randall 2001). One of every 29 women who know they are pregnant report alcohol consumption (Eustace et al. 2003). The toxic effects of in utero alcohol exposure are manifested by a constellation of physical, behavioral, and cognitive abnormalities commonly referred to as fetal alcohol spectrum disorder (FASD) or alcohol-related birth defects (ARBD). In addition to mental retardation, in utero alcohol exposure results in increased rates of miscarriage, reduced birth weight, growth retardation, and teratogenic effects (Jacobson et al. 1998). The incidence of FASD in the general U.S. population ranges from 0.7 to 10 cases per 1,000 live births annually (Eustace et al. 2003), which still is a surprisingly small proportion given the number of children exposed to alcohol during fetal development. The reasons for the low rate of FASD and precise mechanisms causing FASD remain elusive. This article examines the possible contributions of changes in alcohol metabolism during pregnancy and their interaction with maternal nutritional status in determining the degree to which alcohol is toxic to the fetus.

## Rodent Models of Alcohol Consumption in Pregnancy

Pregnancy presents a unique endocrine and metabolic circumstance not found in any other physiologic state with increased nutritional requirements. In addition, the physiologic and metabolic changes of pregnancy can result in altered metabolism of drugs and other chemicals that could result in altered drug efficacy (Ronis and Cunny 2001). Whereas much work has been conducted on the fetal effects of alcohol intake in humans and experimental rodent models, very little work has been published on maternal health related to alcohol intake during pregnancy. Furthermore, changes in maternal metabolism resulting from chronic ethanol intake during pregnancy have not been carefully explored and may have important implications in fetal alcohol exposure.

Rodents serve as good experimental models of human metabolic and endocrine events of pregnancy. By far, most studies of chronic alcohol effects in rodents have been conducted using alcohol-containing liquid diets, such as that developed by Lieber and DeCarli (1989). With few exceptions, laboratory rats, such as the Sprague-Dawley strain, have an aversion to alcohol-containing diets and typically consume 10 to 40 percent fewer calories than control rats given unrestricted access to food (i.e., ad libitum fed), resulting in lower body weight gains among the alcohol-fed rats (Rao and Larkin 1985; Fisher et al. 1997; Keiver et al. 1997). This makes it difficult to distinguish the effects of ethanol alone from the effects of a combination of alcohol and undernutrition. Animals fed the same amount of food as consumed by alcohol-fed rats (i.e., pair-fed controls) typically are used in an effort to account for the undernutrition caused by lower food intake.

Undernutrition is a particular problem in studies of alcohol consumption during pregnancy because of the increased nutrient requirements imposed by the growing fetal–placental unit. Feeding the Lieber–DeCarli diets to pregnant rodents resulted in an 18 to 50 percent reduction in gestational weight gain in pair-fed compared with ad libitum–fed mothers (i.e., dams) (Goad et al. 1984; Weinberg 1985; Keiver et al. 1997). This outcome is directly related to decreased dietary intake and is independent of ethanol consumption (Goad et al. 1984), highlighting the impact of decreased caloric intake of alcohol-containing diets.

To circumvent these problems, investigators have turned to a method of feeding through a tube permanently inserted in the stomach (i.e., intragastric infusion model) in which alcohol is infused as a part of a liquid diet (Badger et al. 1993*a*,*b*). Research by the authors employs the same common procedures of providing diets via feeding tube as used clinically (i.e., total enteral nutrition [TEN]) or intravenously (i.e., total parenteral nutrition) to deliver nutrients at levels recommended by the National Research Council (NRC). Diets fed in this fashion to experimental animals are used to study alcohol/diet interactions that affect endocrine and metabolic systems (Ronis et al. 1991; Badger et al. 1993*a*,*b*). The TEN system provides all the nutrients recommended by the NRC for rats and carefully controls the dose of ethanol.

Diets can either be infused throughout the day (over 23 hours) or through the overnight period to mimic the usual eating patterns of the rat that occur during the dark cycle. The system also is amenable to precise caloric intake, leading to three distinct advantages over methods used in previous studies. First, it eliminates distorted findings resulting from pair-wise feeding regimens or lack of sufficient voluntary dietary intake. Second, it presents a robust, highly reproducible, and precise model to control dietary intake. Third, it makes it easy to estimate exposure to ethanol.

Using the intragastric infusion model, Badger and colleagues (1993*a*,*b*) found that blood ethanol concentrations (BECs) and urine ethanol concentrations (UECs) are highly correlated in cycling (or nonpregnant) and pregnant rats because ethanol equilibrates with body water. This relationship remained significant at gestational day 15 (Badger et al. 2005). Hence, monitoring UECs is an accurate, convenient, and non-invasive method of tracking BECs.

## Alcohol-Metabolizing Enzyme Systems

Alcohol is predominantly broken down in the liver via the alcohol dehydrogenase (ADH) enzyme system. The ADH gene family produces (i.e., encodes) enzymes that metabolize a wide variety of substances (i.e., substrates), including ethanol, vitamin A, other simple alcohols (i.e., aliphatic alcohols), hydroxysteroids, and products of the degradation of fat compounds (i.e., products of lipid peroxidation). At least five different classes (classes I through V) of ADH have been described in humans, and an additional two classes (classes VI and VII) have been found in other species such as the rat and chicken, respectively (Duester et al. 1999). These ADH enzymes vary in how efficiently they break down or oxidize alcohol. ADH1 (an enzyme variant [i.e., isozyme] belonging to the class I ADH) is the most efficient for this substrate, ADH4 is about 10 times less efficient, and ADH3 is nearly inactive toward alcohol. Experiments with mice lacking the gene for ADH1 have revealed that ADH1 contributes to approximately 75 to 90 percent of alcohol metabolism (Deltour et al. 1999). ADH1 is abundant in the liver, although minor amounts are expressed in most tissues. The resulting metabolism byproduct, acetaldehyde, is then rapidly converted to acetate via the aldehyde dehydrogenase (ALDH) enzyme system, which also has three major classes. The oxidation of acetaldehyde is performed most efficiently by ALDH2 (Duester et al. 1998). Other non-ADH systems, such as the enzymes cytochrome P450 and catalase, play a minor role in alcohol metabolism and elimination.

## Alcohol Metabolism and Fetal Alcohol Toxicity

Alcohol toxicity is strikingly evident in a variety of fetal tissues; the brain, skeleton, and liver are the most affected. No single mechanism has been sufficient to account for these varied effects, and it is likely that multiple factors are involved. Researchers have suggested a large number of possible mechanisms, including indirect effects of alcohol on maternal blood supply to the fetus (leading to an alcohol-induced reduced oxygen supply to the fetus); direct effects of alcohol on cell division and cell death; the composition and fluidity of the cell membranes; and an imbalance in the cell’s oxidative status that can lead to cell damage, lipid peroxidation, effects on growth factors (growth hormone, insulin-like growth factor-1 [IGF-1]), and alcohol-mediated inhibition of cell adhesion. However, most studies have concluded that the toxic effects of alcohol are related to the circulating/peak concentrations of alcohol. Two lines of evidence support this.

Because alcohol is freely distributed in total body water, alcohol concentrations in the fetus are comparable with those in the mother. Inhibiting alcohol metabolism (thereby increasing circulating alcohol levels and consequent fetal exposure) in pregnant mice increased the embryotoxicity of alcohol, suggesting that alcohol, rather than its metabolites, is responsible for its toxicity (Ukita et al. 1993). Further evidence comes from human epidemiological studies of FASD incidence and ADH2 polymorphism in a mixed-ancestry population in western Cape Town, South Africa. Because the ADH2*2 gene variant (i.e., allele) encodes the isozymes containing the, β_2_ subunit that oxidizes alcohol 40 times faster in vitro than the, β_1_ subunit (encoded by ADH2*1), people with the ADH2*2 allele have higher rates of alcohol clearance. A comparison of the ADH2*2 allele frequencies of mothers and FASD children revealed that mothers carrying the ADH2*2 allele had a lower incidence of FASD children than mothers without the allele. One of the mechanisms of this protection could be faster metabolism of alcohol attributed to the high-activity enzymes (Eriksson et al. 2001). Therefore, it is evident that factors that increase metabolism and clearance of alcohol during pregnancy can significantly protect the fetus from developing FASD.

## Pregnancy and Alcohol Metabolism

Research with animal models has investigated the effects of pregnancy on alcohol metabolism, the influence of nutrition on this effect, and the potential metabolic mechanisms involved. In one study, pregnant rats given an intravenous dose of 0.75 g/kg of ethanol showed an increased alcohol clearance rate compared with weight-matched nonpregnant rats (Badger et al. 2005). Similar results were observed when alcohol was administered orally. In both experiments, the pregnant rats were able to break down and eliminate alcohol much faster than the nonpregnant rats. Another series of experiments examined whether enhanced clearance of alcohol was limited to a certain period during gestation. When alcohol was administered on days 8, 13, and 19 of pregnancy (in comparison with nonpregnant cohorts), the clearance of alcohol was significantly higher at all time points during gestation compared with nonpregnant rats (Badger et al. 2005).

### Caloric Intake and Alcohol Clearance During Pregnancy

To ensure appropriate nutrition and weight gain for pregnancy, control and alcohol-fed pregnant rats should receive the NRC-recommended 220 kilocalories per kilogram per day (Kcal/kg^3/4^/d),^1^ instead of the 187 Kcal/kg^3/4^/d normally fed to non-pregnant rats. To address whether, this increased intake of calories affects alcohol metabolism pregnant rats received 220 Kcal/kg^3/4^/d, whereas nonpregnant rats received either 187 Kcal/kg^3/4^/d or 220 Kcal/kg^3/4^/d. While pregnant rats showed increased alcohol clearance, increased caloric intake in nonpregnant rats failed to alter the elimination of alcohol, indicating that changes in alcohol metabolism were due to pregnancy and not increased caloric intake (Badger et al. 2005).

### Potential Mechanisms of Faster Alcohol Clearance During Pregnancy

Further studies have attempted to address the specific mechanism that might be involved in the faster alcohol clearance observed during pregnancy. Increased liver ethanol metabolism in pregnant rats appeared not to be associated with changes in liver ADH1 activity (Badger et al. 2005). Consistent with earlier reports, cytochrome P450 CYP2E1 (CYP2E1) activity was actually suppressed by pregnancy. Although mitochondrial ALDH activity was not elevated when measured on the basis of mitochondrial protein, the amount of mitochondria in the liver was markedly increased, resulting in increased overall ALDH activity on a whole-liver basis. Because ADH-dependent oxidation of ethanol is a reversible equilibrium and ALDH oxidation of acetaldehyde to acetate is irreversible, this increase in ALDH activity might accelerate alcohol clearance by speeding up the removal of acetaldehyde. In addition, gastric ADH4 (Class IV ADH) activity was 177 percent higher in pregnant female rats compared with nonpregnant cohorts (Badger et al. 2005). These data are consistent with first-pass metabolism by gastric ADH4 being responsible for a significant part of the pregnancy-induced increase in alcohol clearance.

As previously noted, of the many risk factors for alcohol-related adverse health effects, tissue alcohol concentrations may be the most important. Thus, for a given alcohol intake, ethanol clearance may be the most significant risk factor as higher ethanol clearance would help minimize target tissue concentrations and to a greater extent mitigate fetal alcohol exposure. The data reveiwed above demonstrate that in ad libitum–fed Sprague-Dawley female rats and under the experimental conditions of the TEN rat model, UECs (and thus BECs and tissue ethanol concentrations) are lower in pregnant rats than nonpregnant females given the same dose of ethanol (Badger et al. 2005). Pregnancy provides a substantial degree of protection against ethanol toxicity by clearing ethanol to a greater degree.

## Nutritional Status, Alcohol Metabolism, and Fetal Toxicity

Nutrition plays an important role in assuring a normal pregnancy. Although it has been known for years that there is an important interaction between alcohol and nutrient intake during pregnancy on fetal development, the standard rodent models have not been adequate to study the role of maternal nutrition on the detrimental effects of alcohol on the growing fetus. Poor nutrition can have a number of deleterious effects on pregnancy. Alcohol interferes with nutritional supply to the fetal-placental unit (Dreosti 1993) and nutritionally inadequate maternal diets have been demonstrated to exacerbate the effects of ethanol. Moreover, chronic alcohol consumption can directly or indirectly compromise nutritional status. Using the TEN system, studies have demonstrated that alcohol-induced fetal growth retardation is increased by undernutrition (Shankar et al. 2006). The importance of nutrition on fetal alcohol toxicity was evident when 63 percent of dams showed whole-litter resorptions (i.e. failed to carry their litters to term) when given alcohol (13 g/kg/d) while being undernourished (fed 70 percent of normal caloric intake, 160 Kcal/kg^3/4^/d) compared with none of the dams fed adequate nutrition and the same dose of alcohol. Undernourished pregnant dams also had greater fetal toxicity, as indicated by reduced pup numbers, full-litter resorptions, and reduced birth weight. These data suggested a remarkable alcohol–nutrition interaction (Shankar et al. 2006).

### Nutrition–Alcohol Interactions and Alcohol Metabolism

Several possible mechanisms underlying alcohol–nutrition interactions have been explored. Two principal mechanisms are relevant in this context. First, it is clear that pregnant undernourished rats are unable to break down alcohol as efficiently as pregnant rats that receive adequate nutrition. This puts the fetus at greater risk for the toxic effects of alcohol, as evidenced by the higher amniotic fluid and blood alcohol concentrations. Second, liver ADH1, which is responsible for the majority of alcohol metabolism, also was significantly lower in the undernourished pregnant ethanol-fed dams (Shankar et al. 2006). Although the mechanisms of how nutrition modulates ADH1 levels are not clear, data suggest that undernutrition may lead to alterations in the ADH1 messenger RNA [mRNA]. Several hormones—including growth hormone (GH), thyroid hormone, and androgens that affect ADH mRNA levels—also might be modulated by nutrition. Additionally, proteins involved in the transfer of genetic information from DNA to RNA (i.e., transcription factors), including CCAAT/enhancer–binding protein (C/EBP-β), sterol regulatory element– binding protein (SREBP-1) (He et al. 2002, 2004), and STAT5b have been reported to regulate ADH1 transcription. C/EBPs and SREBPs are transcription factors that play important roles in energy metabolism and can be modulated by nutritional status. Therefore, the hypothesis that under-nutrition impairs alcohol metabolism by decreasing ADH1 levels via altered C/EBPs, SREBP-1c, or STAT5b signaling is worthy of further investigation.

### Global Gene Expression Changes Related to Alcohol–Undernutrition Interactions

Gene expression changes may underlie the combined contributions of alcohol and undernutrition and their interactions in causing fetal toxicity. Using large-scale microarray technology, Shankar and colleagues (2006) examined the changes in the expression of 8,800 genes in the livers of pregnant rats fed alcohol in either adequate or undernourished states. The researchers analyzed the expression of genes to determine how many genes were changed in response to alcohol treatment during pregnancy in the face of either adequate or under-nutrition. The numbers of genes changed in the maternal livers following alcohol treatment in the undernourished group, compared with the adequately fed group given same alcohol dose, differed by approximately 10-fold (369 vs. 37 genes, respectively). Genes involved in cellular metabolism; response to stress and stimulus; protein, carbohydrate, and lipid metabolism; cell-cycle regulation and DNA transcription; cell communication and development; and cell and tissue differentiation were uniquely changed in the undernourished alcohol group (Shankar et al. 2006). Although the expression of a large number of genes was altered, a closer examination of the data showed that IGF-1 was especially decreased in alcohol-fed rats during undernutrition, and several genes known to be regulated by IGF-1 also were altered, making it a candidate for further investigation. For example, activity of the enzyme casein kinase, which is directly increased with IGF-1, was decreased in correlation with IGF-1. Moreover, gene expression of the binding protein (i.e., receptor) GH and IGF binding protein 3 (IGFBP-3) was significantly altered so as to decrease IGF-1 signaling. Detailed analyses of several molecular regulators yielded data consistent with the hypothesis that alcohol-induced fetal growth retardation occurs, for the most part, as a result of impaired placental development and transport of nutrients resulting primarily from disruption of the maternal GH–IGF system (Shankar et al. 2006). The data suggest that undernutrition is a significant risk factor in alcohol- associated fetal growth retardation, and optimal nutritional management during pregnancy may be an effective way to reduce the penetrance of fetal alcohol toxicity in high-risk individuals.

## Footnotes

1 Because caloric requirement best corresponds to body surface area, body weight (kg^3/4^) commonly is used as an approximation to body surface area.
